# Successful Removal of a Foreign Body from the Knee Joint—Anomalous Character of the Same

**Published:** 1852-08

**Authors:** J. Washington Smith

**Affiliations:** East Franklin, Del. Co., N. Y.


ART. II.—*Successful Removal of a Foreign Body from the Knee joint—Anomalous Character of the Same.* By J. WASHINGTON SMITH.

IN June 1851, I was consulted by Mr. R. M., æt. 22, a farm laborer, and otherwise healthy, in regard to a difficulty of the right knee joint. The patient stated that for some months previous something had appeared to "catch," as he termed it, in the joint while using it, causing excruciating pain, followed by inflammation, swelling and weakness of the joint. The peculiar "catch," had only existed for a few months, but the patient says, "the knee has been a little weak and swollen at times, ever since it received a severe wrench, during a 'scuffle' some four or five years previous." Recently he had perceived a loose body, "at times something like a bean," on each side of the patella and at other points, which was freely moveable and would easily slip into the joint. At times it was readily detected, at others it was difficult or impossible. The knee was considerably enlarged, as from chronic thickening of the synovial membrane, and painful to the touch in places.

*Diagnosis.*—After the above history, and manipulating the joint so as to bring the foreign body within reach, I did not hesitate to pronounce it a case of *loose cartilage* within the joint.

The probable cause, nature, course, usual treatment, &c., of such cases were fully explained, and the patient dismissed with the advice to seek older and more experienced counsel, but not to think of an operation, unless it should become more serious, and then not until a trial had been made to "fix" it outside, or else to prevent its escape from within the joint.

At my request the patient called occasionally to report his condition. After a time he acquired such dexterity as to bring the foreign body outside the joint almost at pleasure.

After September, '51, was not able to attend regularly to his occupation; and as the joint was evidently becoming more thickened and stiffened from the continued irritation to which it was subjected, —efforts were made to "fix" the cartilage (as I still supposed it), by pushing it as far away from the joint as possible, in one of the synovial pouches, by the side of the patella, but it was found upon repeated trials that it could not be confined *in situ* by any amount of pressure which could be borne, so as to allow any motion of the joint. Efforts were also made to confine it *within* the joint, but with only partial success, while the cause of irritation was sure to be at work.

There now appeared but one other chance, viz., an operation, and this, owing to the state of the joint, it must be confessed, was not very promising. The almost certain result of the case as it *was*, together with the *probable* and *possible* consequence of an operation for the removal of the offending body, were again fully and fairly stated to the patient and friends. Dr. Almiron Fitch of Delhi, distinguished for ability and experience in his profession, was also consulted and coincided fully in the opinion given.

*Operation.*—In March, '52, I was requested to remove the foreign body by operation. Having enjoined rest, low diet, and occasional purgatives during the preceding week, on the 10th inst. I proceeded to remove it—Dr. Fitch, from whom I received valuable assistance, kindly consented to be present. Some delay and difficulty were experienced in fixing the body externally to the joint, and in the position desired, viz., upon the inner condyle, without occasioning too much motion of the joint. The integuments were then drawn tensely forward, (nearly one inch,) while the cartilage(?) was firmly fixed by the fingers of an assistant. A longitudinal incision, three-fourths of an inch in length, was then carried through the integuments, directly upon the cartilage(?) but as it could not readily be removed, a second incision was made so as slightly to enlarge the opening, when, with a tenaculum, it was removed with some difficulty, owing to the thickened state of the integuments and the unexpected *hardness* of the supposed cartilage. In form it much resembled an almond,—was eight lines in length, six broad and four in thickness,—and was completely enveloped in healthy appearing cartilage. Its substance was evidently *osseous* or *calcareous* but unfortunately (and contrary to agreement,) it passed out of my possession before I had an opportunity of subjecting it to any chemical or microscopic tests.

The hæmorrhage was slight and soon ceased, when plasters were applied so as to nicely close the incision; a compress was carefully adjusted so as to securely close the *valvular* opening, and over these a figure of 8 bandage; the whole being completed by a long splint to the outside of the limb, secured by roller, except over the knee, nearly preventing all motion of the joint. Water was applied several times a day and was the only application. There was a slight exudation of serum, but none of synovia. The diet was light for a few days, and small occasional doses of pil. cath. comp. and pulv. jalap. comp. were given to procure the regular evacuation of the bowels. Union was secured partly by first intention, and partly by what is called by Macartney the *modelling* process, *i.e.*, without suppuration. The case was closely watched, and there was no inflammation at any time. After a few days the patient sat up part of the time, but the plasters and splint were not dispensed with until the thirteenth day, when the wound appeared completely united. A compress and figure 8 bandage were continued, and directions given to use and flex the limb but slightly; but from that time he began and continued to go about. By the 15th April there was only a slight weakness and some stiffness upon flexing to an acute angle. At the end of six weeks he was attending to his ordinary occupation, and was able to join, as he was wont to do, "in the giddy mazes of the dance."

*Remarks.*—The operation by subcutaneous incision, as proposed by Prof. Syme and M. Goyrand, was hardly practicable, owing to the thickened state of the integuments, though it would in most cases greatly lessen the danger of subsequent inflammation. Wounds penetrating the cavities of joints, especially the larger, have ever and justly been the dread of surgeons, and the common result an opprobrium to the healing art.

This case is a striking illustration of the importance of *perfect rest* (of the joint) and a simple but not *too* antiphlogistic treatment in wounds and injuries of joints; though in regard to the latter the previous habits should be our guide. Here there was evidently a *want* of action, and though the diet was light for only a few days, had it been more generous, I am satisfied complete union by first intention would have taken place, a most desirable result in wounds of this nature.

The history will doubtless satisfy some minds that a *fragment* of bone was nearly or quite detached at the time of the injury mentioned; but to my mind it is not so conclusive. A section of one end of the foreign body revealed a hard, friable substance, more resembling calculus. But was a calculus ever completely covered with healthy looking cartilage? If so, it is a most remarkable provision of nature to prevent injury to the joint. May not a peculiar abnormal state of the synovial membrane, from any cause, give rise to an adventitious growth or deposit in the synovia as well as in the other fluids of the body? Loose cartilages and calculous concretions in joints, are not very unusual, but I do not recollect any account of a foreign body in the cavity of a joint, similar to the present.

The patient consulted different surgeons, to whom he stated the symptoms and diagnosis; but it is a curious fact that none of them at the time ever detected the foreign body, (though part were satisfied of its existence,) and at one time, believing it a case of chronic synovitis, counter irritants and ung. iod. comp. were freely applied with the view of procuring absorption!

East Franklin, Del. Co., N. Y., July, 1852.

